# An Optimized Approach to Channel Modeling and Impact of Deteriorating Factors on Wireless Communication in Underground Mines

**DOI:** 10.3390/s21175905

**Published:** 2021-09-02

**Authors:** Fawad Javaid, Anyi Wang, Muhammad Usman Sana, Asif Husain, Imran Ashraf

**Affiliations:** 1 College of Communication and Information Engineering, Xi’an University of Science and Technology, Xi’an 710054, China; fawaddayyal@yahoo.com (F.J.); asifarif55@yahoo.com (A.H.); 2 College of Computer Science Technology, Xi’an University of Science and Technology, Xi’an 710054, China; m.usman@uog.edu.pk; 3 Department of Information and Communication Engineering, Yeungnam University, Gyeongsan 38544, Korea

**Keywords:** underground wireless communication, EM wave propagation, hybrid channel modeling, mine waveguide model, path loss

## Abstract

The environment of underground coal mines has challenging properties that makes this zone inadaptable for a stable communication system. Additionally, various deteriorating physical parameters strongly affect the performance of wireless networks, which leads to limited network coverage and poor quality of data communication. This study investigates the communication capability in underground coal mines by optimizing the wireless link to develop a stable network for an underground hazardous environment. A hybrid channel-modeling scheme is proposed to characterize the environment of underground mines for wireless communication by classifying the area of a mine into the main gallery and sub-galleries. The complex segments of mine are evaluated by categorizing the wireless links for the line-of-sight (LOS) zones and hybrid modeling is employed to examine the characteristics of electromagnetic signal propagation. For hybrid channel modeling, the multimode waveguide model and geometrical optic (GO) model are used for developing an optimal framework that improves the accessibility of the network in the critical time-varying environment of mines. Moreover, the influence of various deteriorating factors is analyzed using 2.4 GHz to 5 GHz frequency band to study its relationship with the vital constraints of an underground mine. The critical factors such as path loss, roughness loss, delay spread, and shadow fading are examined under detailed analysis with variation in link structure for the mine.

## 1. Introduction

Underground coal mines have a complex and unfavorable environment with inappropriate conditions to establish a stable communication system. Traditional communication techniques are less sophisticated for underground coal mines and conditions including uneven structure, rough walls, large mining equipment, non-uniform permittivity, and irregular geometry [1]. The underground roadway is a closed limited space, made up of rocky walls that affect the transmission of electromagnetic waves (EM) and produces high attenuation [2].

Ores inside the mines are fixed in the ground, but available in variable chunks over a long distance; hence, the mining activities are spread over the entire mine. Therefore, to cover the whole mine is a complex task for a communication system. The existing underground mine system provides limited monitoring of sites, and cables are the major source of information exchange [3]. As wires become damaged, it destroys the whole network and paralyzes the entire system which disturbs the safety of the mines. Similarly, power transmission is another serious matter to consider, as mines consist of explosive materials and high-power signals can cause disaster [4]. Predominantly, damages to network infrastructure from accidents in underground mines lead to link deterioration and communication failure. Consequently, the safety of workers is at stake. Therefore, an efficient and reliable communication system is required to ensure their safety. A system is required that can bear disasters and provide a robust tracking methodology. A stable communication framework does not only help to save work process time of machinery, but it also helps to transfer information quickly between a monitoring point and other underground units. As a result, it speeds up the rescue work in the case of accidents and natural disaster [5]. As well as the underground communication assistance and location estimation, a stable wireless system is useful for the control of mining equipment, remote monitoring, real-time access for operating information, and data gathering from a variety of sensors for underground mines. Therefore, a stable communication system is required to ensure smooth operations in underground mines and better safety.

**Figure 1:**
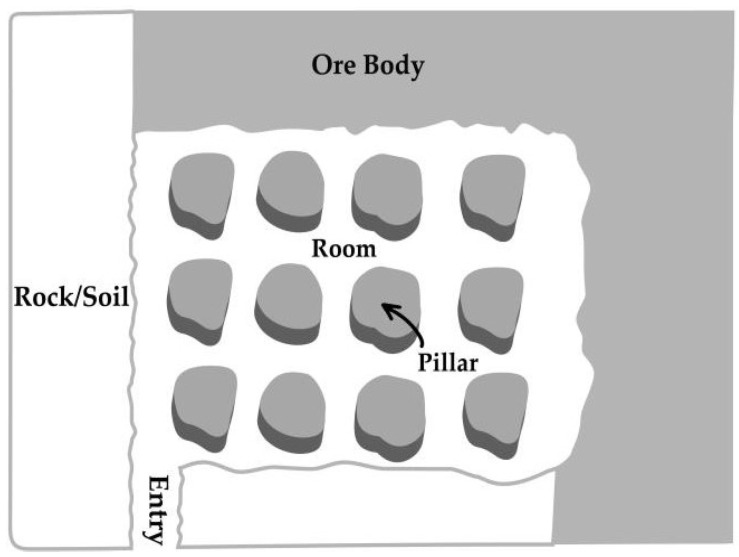
Room-and-pillar architecture of coal mines.

Concerning the significance of underground communication, although a large body of research exists for safe communication, several problems require immediate attention. One of the primary concerns is a safe and efficient wireless communication system to realize fast-track flexible sensor access, and larger coverage to underground monitoring areas [1,6]. Physical structure and a sensitive medium of underground mines plays an important role in signifying the characteristics of signal propagation. Therefore, this research evaluates the influence of multiple deteriorating factors on wireless links by employing the optimized channel-modeling approach. A hybrid channel model scheme is used by classifying the different zones of a mine to examine the characteristics of electromagnetic signals. The main entrance of a mine is considered to be the mine gallery and the multiple branches are represented as sub-galleries. A sub-gallery comprises of a room-and-pillar structure and the remaining ore body for the extraction of minerals, as shown in Figure 1. The channel modeling for both nominated zones is conducted according to their environment and the signal-received power is analyzed by categorizing the LOS zones to inspect the performance of wireless links in complex areas. In summary, the proposed system makes the following contributions:

- This study investigates the influence of several deteriorating factors on a wireless link using the 2.4 GHz to 5 GHz frequency band in underground coal mines.
- The characteristics of electromagnetic signal propagation are investigated for complex segments of a mine using line-of-sight (LOS) links.
- A hybrid channel-modeling approach is proposed for communication in underground coal mines using a multimode waveguide model and geometrical optic (GO) model.
- Performance evaluation of the proposed approach is done using several critical factors such as path loss, received power loss, delay spread, and shadow fading.

The rest of the paper is organized in the following manner. Section 2 presents a brief overview of conventional communication techniques for mines and potential challenges for underground communication. A review of existing channel models for communication in underground mines is given in Section 3. A description of the proposed hybrid channel model is presented in Section 4. Section 5 contains the results and discussion, and the conclusion is given in Section 6.

## 2. Overview of Conventional Communication Techniques and Potential Communication Challenges in Underground Mines

This section briefly describes the conventional communication techniques and potential challenges in underground mines that complicates the development of an efficient channel model for wireless communication.

### 2.1. Conventional Communication Channels

The underground communication network (UCN) consists of wireless or wired devices that are either placed in a bounded open space of tunnels or completely buried under dense soil [7]. A brief discussion of existing conventional communication techniques that have been applied in the underground mines is given here.

#### 2.1.1. Through-Wire and Hybrid Scheme

In the harsh environment of underground mines, a fixed infrastructure of wires can always be the source of problems for mine workers. Lacking a stable wireless communication framework, signal transmission is carried out with the aid of cables called through-the-wire (TW) communication [3]. Coaxial cables and the optical fibers are typically used in mines for data transmission, but that does not provide network accessibility in all areas of a mine [8]. For the protection of a wired system, various schemes have been introduced including the deployment of pipes and placing cables via borehole connections to the mainline.

**Figure 2:**
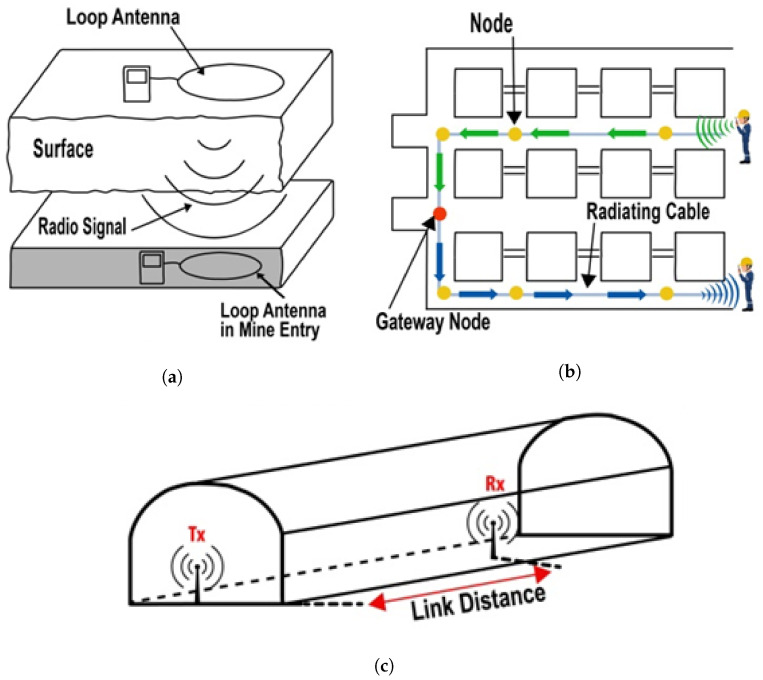
Pictorial presentation of conventional communication methods (**a**) through-earth, (**b**) through-wire, and (**c**) through-air communication methods.

In some cases, hybrid systems are used that consist of leaky feeder cables that radiate signals for data reception [9]. However, such techniques require higher maintenance effort, and an independent stable wireless network is still required in mines. The fundamental methodologies used in conventional communication schemes are shown in Figure 2.

#### 2.1.2. Through-Earth (TE) and Through-Air (TA)

The through-earth (TE) scheme is data transmission using the propagation of EM signals through the surface of the earth. These signals penetrate in depth through the multiple layers of underground mines; however, such propagation weakens the power of EM signals [10]. Even so, the range of signal transmission can be improved using particular frequencies of less than 10 kHz [11]. The attenuation of EM signals in the TE scheme depends on multiple factors such as antenna size, transmit power, bandwidth, and noise levels, etc. TE operates normally between 90 Hz to 9 kHz, and it is necessary to launch EM signals using large loop antennas [12]. On the other hand, through-the-air (TA) represents the communication framework that uses wireless links through free space inside mines for data transmission. The major problem in this approach is its adaptability in a mine’s critical environment. Physical characteristics of a mine, inefficient channel modeling, and mining material create a high-level scattering for propagated signals, which reduces the efficiency of this approach [13].

### 2.2. Potential Challenges in Underground Mines

#### 2.2.1. Extreme Path Loss and Multipath Fading

Path loss is one of the major concerns for propagated signals in underground mines. Communication channels share several characteristics in the critical environment of mines, which leads to difficulty in reliable operation. As the signal propagates away from the transmitter, extreme path loss is observed in the gallery of a mine due to irregular geometric effects [6]. The link distance has a direct relationship with the attenuating factors and inverse relationship with propagated signal power [14]. The dielectric medium of underground mines changes its properties with a change in temperature and moisture, which leads to signal absorption [15]. Reflecting surfaces of underground mines and mining equipment used for mineral extraction are the prime sources of disruption for propagated signals. These obstructions produce a multipath propagation effect due to the reflection of signals, which leads to fading and fluctuations of the propagated signal [16]. When the signal travels through multiple routes and ends up at a particular receiver with a specific time delay, this effect reflects the dispersive properties of the channel, which leads to induction of the delay spread [17].

#### 2.2.2. Rapid Time-Varying Channels and Propagation Velocity/Delay

Environmental channel characteristics in mines change irregularly, which complicates the design of an efficient channel model. Frequent movement of convenient communication equipment along with variations in the prime channel of mines leads to the production of Doppler frequency shifts and fast signal strength fluctuations [18]. Signal propagation characteristics in a terrestrial environment are very much different from a compact reflected environment of underground mines; the signals face a lot of hindrance from the dielectric medium of mines. Variation in properties of such mediums and non-homogeneous materials can generate a propagation delay effect for transmitted signals [19]. Power cabling, electric machinery, and other mining appliances that are used by underground communication can create some noise in particular frequency bands, which corrupts data bits and adds interference during the signal propagation [20].

#### 2.2.3. Gaseous Hazards and Limited-Line-of-Sight Problems

One of the main factors that causes interruption of communication signals is the eruption of flammable gases. A risk of gas blast increases when the concentration of such gases exceeds a particular threshold. Therefore, continuous evacuation is required to decrease the probability of any disaster. Moreover, the explosion or any disaster can affect the propagation characteristics of EM signals and leads to a complete link failure [21]. LOS links in such a critical environment improve the quality of wireless communication because waves can propagate directly towards the receiver, rather than around the corner or through materials, as both cases produce high attenuation [22]. However, in underground mines, a congested structure of room-and-pillar and tunnel blockages restrict LOS communication.

## 3. Review of Existing Channel Models for Underground Mines

This section describes various analytical models for characterizing wireless propagation in underground mines. Existing models for channel analysis used in underground mines are briefly discussed, and a summary of their benefits and drawbacks is given in Table 1.

### 3.1. Single-Mode Waveguide Model

Tunnel structure in underground mines can guide EM waves throughout their path and this type of EM wave propagation has been modeled as a waveguide [23]. In the single-mode model, the distributed field of EM waves is examined by adding propagated modes on the excitation plane with the help of Maxwell equations. The single-mode waveguide model becomes the base for modeling in underground tunnels, especially for evaluating path loss in lower frequencies of the ultra-high frequency (UHF) band [7]. However, this model is considered valid only for the dominant mode and for evaluating the propagation loss in the far-field region [14]. Several efforts have been made for the correction of weak areas by modifications, but no model is valid for path loss in the near-field region. Moreover, this model fails to predict accurate results on higher frequencies and does not cover the surface roughness of tunnel walls [24].

### 3.2. Geometrical Optic Model

For smaller-wavelength operations in underground mines, the GO model is useful to estimate the characteristics of the propagated signal in complex environments. Unlike the single-mode waveguide model, it can be used to investigate branches of mines for signal propagation. It can provide accurate results when the dimensions of the environment are larger than its wavelength. This means that it can satisfy the higher UHF-band frequencies for propagation loss in underground mines. The use of this model is based on optical ray theory, where EM waves used for signal transmission are considered to be optical rays [24]. The distributed fields of propagated signals can be computed with the addition of replicated images produced by the interior surfaces of a tunnel. The GO model can also be applied to analyze an occupied mine, but this model is not valid for characterizing lower frequencies. Moreover, it does not cover diffraction loss in mines. Another drawback is its computational complexity on high link distance [14,25].

### 3.3. Multimode Waveguide Model

In the environment of underground mines, the multimode model can be used to estimate the signal propagation characteristics for the near and far regions. This model is valid for multiple frequencies, and the operating frequency in this model can be higher than the cut-off frequencies of propagation by increasing communication frequency [26]. The propagation loss analysis concerning the root mean square (RMS) delay spread can be estimated using the multimode waveguide model. However, this model is not friendly for complex environments in underground mines, having multiple branches and being incapable of estimating a mine’s roughness parameter [27].

### 3.4. Full-Wave Model

The full-wave model is an effective scheme to estimate channel characteristics for underground mines. This approach is considered valid for analyzing the degrading effects of reflection, diffraction, and refraction in tunnels with a complete solution for signal propagation in complex areas [28]. It is considered an alternative approach to compute Maxwell equations with the aid of a numerical technique that comprises the Finite-Difference Time Domain (FDTD). The weakness of this model is the computational complexity of the FDTD beyond the normal capacity for large-size tunnels and higher UHF-band frequencies [7].

## 4. Proposed Methodology of Hybrid Channel Modeling

**Figure 3:**
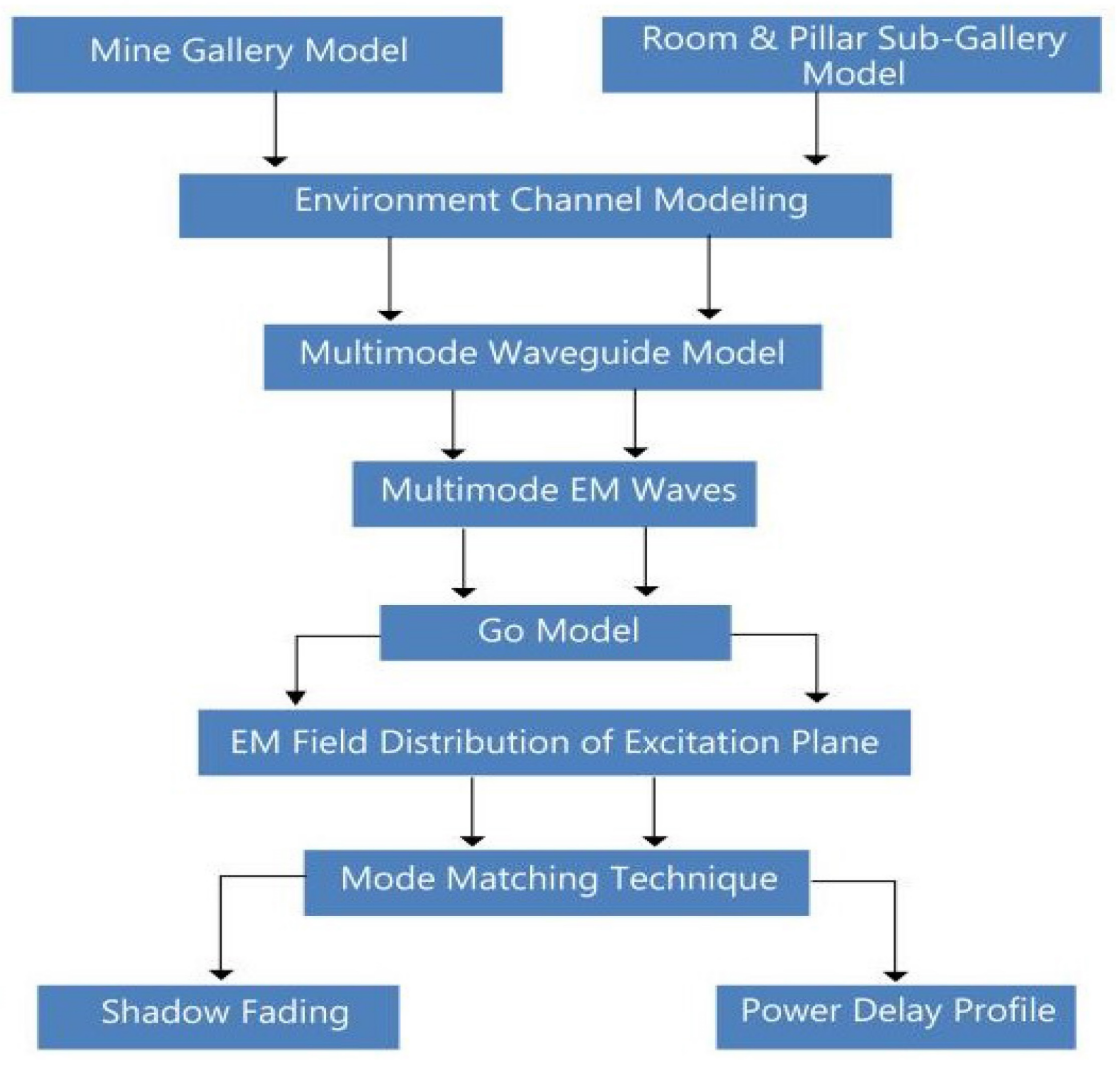
Flow diagram of proposed hybrid channel-modeling scheme for mines.

This section describes the development of a hybrid channel-modeling scheme by distinctly using the multimode waveguide and GO model for underground mines. The complete flow structure of channel modeling is shown in Figure 3. The multimode waveguide model is used to characterize electromagnetic waves, and the basic modes of this model are the key explanations to Maxwell expressions, which particularize the existence of specific EM signals in the critical environment of mines. However, the major problem is its reliance on mode intensity and estimation from the excitation plane, which cannot be resolved with the multimode model [24,29]. Therefore, the GO model is used to facilitate the cross-section area of the transmitter, as it is useful to analyze the EM distributed field.

**Figure 4:**
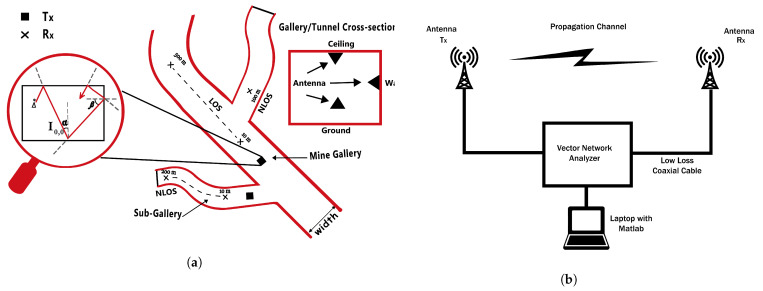
Layout of an underground coal mine, (**a**) Underground mine galleries, (**b**) Channel measurement setup.

This research categorizes the areas of the mine into different categories. The tunnel of the underground mine is considered to be the mine gallery, and its branches are represented as sub-galleries, as shown in Figure 4. The interior architecture of the sub-gallery comprises the room-and-pillar structure, which resembles the free-space-congested terrestrial environment of high buildings.

### 4.1. Gallery Model for Underground Mine

It is very important to consider the structure of the mine before developing its mathematical model. Although mines consist of different portions, in this case the general cross-section area for signal propagation is in rectangular form [30]. In this study, the width of the assumed mine gallery is 2a, and the height is considered to be 2b, and the origin is in the center using the Cartesian coordinate framework. The complex electrical parameters for the mine gallery are given below in (1)–(3):

(1) $$\begin{array}{c} k_{v}=\epsilon_{0}\epsilon_{v}+\frac{\sigma_{v}}{j2\pi f_{0}} \end{array}$$

(2) $$\begin{array}{c} k_{h}=\epsilon_{0}\epsilon_{h}+\frac{\sigma_{h}}{j2\pi f_{0}} \end{array}$$

(3) $$\begin{array}{c} k_{a}=\epsilon_{0}\epsilon_{a}+\frac{\sigma_{a}}{j2\pi f_{0}} \end{array}$$

where $\epsilon_{v}$, $\epsilon_{h}$, $\epsilon_{a}$ and $\epsilon_{0}$ denote relative permittivity parameters for the vertical structure of sidewalls, horizontal floor, air, and vacuum space in the tunnel, respectively. Similarly, $\sigma_{v}$, $\sigma_{h}$ and $\sigma_{a}$ represents the conductivity parameters for vertical side boundaries, horizontal top/bottom, and free space of air, respectively. The $f_{0}$ denotes the central frequency and the permeability parameter $\mu_{0}$ is considered to be the same for all areas. The relative electrical parameters $\bar{k_{v}}$ and $\bar{k_{h}}$ are considered to generate a brief expression where $\bar{k_{v}}=k_{v}/k_{a}$ and $\bar{k_{h}}=k_{h}/k_{a}$.

### 4.2. Electromagnetic Propagation in a Mine Gallery

The transmission of EM signals in the mine can be observed in the form of a superposition of various modes with varied distributed field patterns and attenuation coefficients. The complete distributed EM field patterns of every mode can be derived by sorting Maxwell’s expression in the form of Eigen-function [31], as given in (4):

(4) $$E_{m,n}^{eign}\left(x,y\right)≅sin\left(\frac{m\pi }{2a}x+\varphi_{x}\right)cos\left(\frac{n\pi }{2b}y+\varphi_{y}\right)$$

where the values for $\varphi_{x}$ and $\varphi_{y}$ are calculated using

(5) $$\left\{\begin{array}{cc} \varphi_{x}=0 & \mathrm{if}\;m\;\mathrm{is}\;\mathrm{even}\\ \varphi_{x}=\pi /2 & \mathrm{if}\;m\;\mathrm{is}\;\mathrm{odd}\\ \varphi_{y}=0 & \mathrm{if}\;n\;\mathrm{is}\;\mathrm{odd}\\ \varphi_{y}=\pi /2 & \mathrm{if}\;n\;\mathrm{is}\;\mathrm{even} \end{array}\right.$$

The distribution field is calculated against any position (x,y,z) in the mine by the addition of particular EM fields of each substantial mode, and the expression is given below in (6):

(6) $$E^{Rx}\left(x,y,z\right)=\sum_{m=1}^{\infty }\sum_{n=1}^{\infty }C_{mn}E_{m,n}^{eign}\left(x,y\right)e^{-(\alpha_{mn}+j\beta_{mn})z}$$

where $C_{mn}$, $\alpha_{mn}$ and $\beta_{mn}$ represent intensity of modes, attenuation and coefficient of phase shift, respectively [32,33]. The expressions for the coefficient of attenuation and phase shift are given below

(7) $$\begin{array}{c} \alpha_{mn}=\frac{1}{a}{\left(\frac{m\pi }{2ak}\right)}^{2}Re\frac{\bar{k_{v}}}{\sqrt{\bar{k_{v}}-1}}+\frac{1}{b}{\left(\frac{n\pi }{2bk}\right)}^{2}Re\frac{1}{\sqrt{\bar{k_{h}}-1}} \end{array}$$

(8) $$\begin{array}{c} \beta_{mn}=\sqrt{k^{2}-{\left(\frac{m\pi }{2a}\right)}^{2}-{\left(\frac{n\pi }{2b}\right)}^{2}} \end{array}$$

The waveguide model declares the lower-order mode in the environment of underground mine as $C_{11}=1$ and $C_{mn}=0$ if (m,n)≠(1,1) but multiple modes exist in the near zone of the transmitting antenna with particular intensities. To find the field distribution and mode intensity $C_{mn}$ on the excitation plane, the GO model is used in the current study.

### 4.3. Electromagnetic Field Analysis in the Mine Gallery

The entire distribution field on the excitation plane is obtained by summing up the contribution of the source and total reflected images. In a rectangular-shaped mine gallery, this geometry exhibits the protocol that vertical walls reflect the image $I_{pq}\left|p\right|$ times and horizontal ceilings or floors reflect |q| times.

Assume that the incident angle for the horizontal floor or ceiling is α and the incident angle for vertical walls is β, as shown in Figure 4 with the basic design of the mine. If the transmitter is set at coordinates $\left(x_{0},y_{0},0\right)$, and the observant node is located at (x,y,z), then the net field on an observant node can be considered to be the summation of all rays, and the field at the transmitter node $E_{0}$ can be calculated as

(9) $$E^{Rx}\left(x,y,z\right)=E_{0}\sum_{p=-\infty }^{\infty }\sum_{q=-\infty }^{\infty }\left[\frac{exp(-jkr_{p,q})}{r_{p,q}}\right]S{\left(\bar{k_{v}}\right)}^{\left|p\right|}R{\left(\bar{k_{h}}\right)}^{\left|q\right|}$$

where $R(\bar{k_{h}})$ and $S(\bar{k_{v}})$ are reflection coefficients for horizontal and vertical parameters, respectively, $r_{p,q}$ denotes the distance between the receiver and image $I_{p,q}$, as described in Equations (10) and (11). The $r_{p,q}$ is given in Equation (14), where a +ve sign represents the scenario when *p* or *q* is even and a −ve sign for *p* or *q* represents the odd.

(10) $$R\left(\bar{k_{h}}\right)=-exp\left(\frac{-2sin\alpha )}{\sqrt{\bar{k_{h}}-1}}\right)$$

(11) $$S\left(\bar{k_{v}}\right)=-exp\left(\frac{-2sin\beta )}{\sqrt{\bar{k_{v}}-1}}\right)$$

The parameters $R(\bar{k_{h}})$ and $S(\bar{k_{v}})$ can be transformed into simplified equations, using the following

(12) $$R\left(\bar{k_{h}}\right)=-exp\left(\frac{-2}{\sqrt{\bar{k_{h}}-1}}\frac{|2qb\pm y_{0}-y|}{r_{p,q}}\right)$$

(13) $$S\left(\bar{k_{v}}\right)=-exp\left(\frac{-2\bar{k_{v}}}{\sqrt{\bar{k_{v}}-1}}\frac{|2pa\pm x_{0}-x|}{r_{p,q}}\right)$$

(14) $$r_{(}p,q)=\sqrt{\left({\left(2pa\pm x_{0}-x\right)}^{2}+{\left(2qb\pm y_{0}-y\right)}^{2}+z^{2}\right)}$$

The ray sum is reorganized from Equation (9) and split into four parts, as given below

(15) $$\begin{array}{c} E^{Rx}\left(x,y,z\right)=\sum_{p,q=-\infty }^{\infty }f\left(4qa+x_{0}-x,4pb+y_{0}-y\right)\\ +\sum_{p,q=-\infty }^{\infty }f\left(4qa+x_{0}-x,4pb+2b-y_{0}-y\right)\\ +\sum_{p,q=-\infty }^{\infty }f\left(4qa+2a-x_{0}-x,4pb+y_{0}-y\right)\\ +\sum_{p,q=-\infty }^{\infty }f\left(4qa+2a-x_{0}-x,4pb+2b-y_{0}-y\right) \end{array}$$

And the function f(u,v) is calculated using

(16) $$\begin{array}{c} f\left(u,v\right)=E_{0}.\frac{exp(-jk\sqrt{u^{2}+v^{2}+z^{2}})}{\sqrt{u^{2}+v^{2}+z^{2}}}{\left(-1\right)}^{p(v)+q(u)}\\ .exp\left[\frac{-2}{\sqrt{u^{2}+v^{2}+z^{2}}}\left(\frac{\left|v|p(v\right)}{\sqrt{\bar{k_{h}}-1}}+\frac{\left|u\right|\bar{k_{v}}q\left(u\right)}{\sqrt{\bar{k_{v}}-1}}\right)\right] \end{array}$$

The parameters p(v) and q(u) need to be transformed from discontinuous to continuous functions for mode-matching purpose, as given below

(17) $$p\left(v\right)=\frac{\left|v\right|}{2b}\;;\;q\left(u\right)=\frac{\left|u\right|}{2a}$$

It is noteworthy to point out that every segment of Equation (15) reflects the periodic function of 4a and 4b. The first part is entertained first, and the sum is transformed into the following expression (18) under a two-dimensional Poisson summation expression [34]

(18) $$\begin{array}{c} \sum_{p,q=-\infty }^{\infty }f\left(4qa+x_{0}-x,4pb+y_{0}-y\right)=\\ \frac{1}{4a}\frac{1}{4b}\sum_{m=-\infty }^{\infty }\sum_{n=-\infty }^{\infty }F_{1}\left(m,n\right)e^{j\frac{m\pi }{2a}x}e^{j\frac{n\pi }{2b}y} \end{array}$$

The coefficient $F_{1}\left(m,n\right)$ denotes Fourier transform of the function $f(x_{0}-x,y_{0}-y)$ for the first part given in (15):

(19) $$F_{1}\left(m,n\right)=\int \int_{-\infty }^{\infty }f\left(x_{0}-x,y_{0}-y\right)e^{-j\frac{m\pi }{2a}x}e^{-j\frac{n\pi }{2b}y}dxdy$$

The Saddle-point methodology is used to obtain the result of integration, as the study is focused on the field $E^{Rx}\left(x,y,z\right)$ on the excitation plane with z=0. Hence, the parameter $F_{1}\left(m,n\right)$ can be stated as

(20) $$F_{1}\left(m,n\right)≅E_{0}\frac{\pi }{\sqrt{1-{\left(\frac{m\pi }{2ak}\right)}^{2}-{\left(\frac{n\pi }{2bk}\right)}^{2}}}e^{-j(\frac{m\pi }{2a}x_{0}+\frac{n\pi }{2b}y_{0})}$$

Similarly, the sum of complex modes in Equation (18) can be obtained by converting the first part of the ray sum in Equation (15). Consequently, the Poisson summation expression can be applied on Equation (15) and $F_{2}\left(m,n\right)$, $F_{3}\left(m,n\right)$, $F_{4}\left(m,n\right)$ coefficients can be simplified using a Saddle-point approach. Then, the field on the excitation plane can be achieved using

(21) $$\begin{array}{c} E^{Rx}\left(x,y,0\right)=\frac{1}{4a}\frac{1}{4b}\sum_{m=-\infty }^{\infty }\sum_{n=-\infty }^{\infty }[F_{1}\left(m,n\right)+F_{2}\left(m,n\right)+F_{3}\left(m,n\right)+\\ F_{4}\left(m,n\right)]e^{j\frac{m\pi }{2a}x}e^{j\frac{n\pi }{2b}y}\\ E^{Rx}\left(x,y,0\right)=\sum_{m=-\infty }^{\infty }\sum_{n=-\infty }^{\infty }\frac{E_{0}\pi }{16ab\sqrt{1-{\left(\frac{m\pi }{2ak}\right)}^{2}-{\left(\frac{n\pi }{2bk}\right)}^{2}}}e^{j\frac{m\pi }{2a}x}e^{j\frac{n\pi }{2b}y}\\ (e^{-j\frac{m\pi }{2a}x_{0}}e^{-j\frac{n\pi }{2b}y_{0}}+e^{j\frac{m\pi }{2a}x_{0}-m\pi }e^{j\frac{n\pi }{2b}y_{0}-n\pi }-e^{j\frac{m\pi }{2a}x_{0}}e^{j\frac{n\pi }{2b}y_{0}-n\pi }-\\ e^{j\frac{m\pi }{2a}x_{0}-m\pi }e^{j\frac{n\pi }{2b}y_{0}})\\ E^{Rx}\left(x,y,0\right)=\sum_{m=1}^{\infty }\sum_{n=1}^{\infty }\frac{E_{0}\pi }{ab\sqrt{1-{\left(\frac{m\pi }{2ak}\right)}^{2}-{\left(\frac{n\pi }{2bk}\right)}^{2}}}\\ \sin\left(\frac{m\pi }{2a}x_{0}+\varphi_{x}\right)\cos\left(\frac{n\pi }{2b}y_{0}+\varphi_{y}\right)\sin\left(\frac{m\pi }{2a}x+\varphi_{x}\right)\cos\left(\frac{n\pi }{2b}y+\varphi_{y}\right) \end{array}$$

Equation (21) is the actual summation form of Eigen-functions of all propagation modes given in Equation (4) and the Eigen-function of each mode represents the mode intensity $C_{mn}$ as given in Equation (22)

(22) $$C_{mn}=\frac{E_{0}\pi }{ab\sqrt{1-{\left(\frac{m\pi }{2ak}\right)}^{2}-{\left(\frac{n\pi }{2bk}\right)}^{2}}}\sin\left(\frac{m\pi }{2a}x_{0}+\varphi_{x}\right)\cos\left(\frac{n\pi }{2b}y_{0}+\varphi_{y}\right)$$

The field can be computed at a point within the mine gallery by substituting Equations (4), (7), (8) and (22) into Equation (6). Let $P_{t}$ be the power of the transmitted signal, $G_{t}$ be the gain of transmitter and $G_{r}$ be the antenna gain of the receiver; the computed received power at the coordinate (x,y,z) can be calculated using

(23) $$P_{r}\left(x,y,z\right)=P_{t}G_{t}G_{r}{\left(\frac{1}{E_{0}}\sum_{m,n}C_{mn}E_{m,n}^{eign}\left(x,y\right)e^{-(\alpha_{mn}+j\beta_{mn})z)}\right)}^{2}$$

### 4.4. Power Delay Profile of Mine’s Gallery

For wideband transmitted signals, distortion is caused by dispersion in the environment of underground mines which can be a source of inter-symbol interference (ISI). This work investigates the power delay profile (PDP) by characterizing the propagation channel. Let a signal s(t) have the bandwidth *B* and central frequency $f_{0}$, generating an expression $f\in (f_{0}-B/2,f_{0}+B/2)$, so the actual signal can be obtained by the addition of each sine wave that belongs to this band.

The strength of each signal can be obtained by taking a Fourier transform of signal s(t) and the resultant S(f) which is an even function of frequency *f* as described below

(24) $$s\left(t\right)=\int_{f_{0}-B/2}^{f_{0}+B/2}S\left(f\right)2\cos\left(2\pi ft\right)df$$

The mode intensity parameter $C_{mn}\left(f\right)$, EM field distribution $E_{m,n}^{eign}\left(x,y,f\right)$, attenuation coefficients $\alpha_{mn}\left(f\right)$ and phase-shift coefficients $\beta_{mn}\left(f\right)$ is transformed into frequency-dependent functions and the variation can be obtained with the frequency in the transmission delay of any specific mode. The transmission delay function $EH_{mn}\left(f\right)$ can be computed by $\tau_{mn}\left(f\right)=z/v_{mn}\left(f\right)$, where $v_{mn}\left(f\right)$ denotes the group velocity as given below

(25) $$v_{mn}\left(f\right)=c\sqrt{1-{\left(\frac{c\sqrt{{\left(\frac{m\pi }{2a}\right)}^{2}+{\left(\frac{n\pi }{2b}\right)}^{2}}}{2\pi f}\right)}^{2}}$$

As shown in Equation (25), the group velocity depends on frequency and mode order. Different frequency signals from the same mode can have different propagation delays. Conversely, multiple modes of identical frequency exhibit different propagation delays. Dispersal among the frequencies and modes are considered for computing power delay profile. The relative received power $P_{WB}$ for a wideband signal can be obtained by the summation of the entire contribution from all particular modes and frequency elements for a certain time *t* at any position (x,y,z) using

(26) $$\begin{array}{c} P_{WB}\left(x,z,t\right)=P_{t}G_{t}G_{r}\{\frac{1}{E_{0}}\sum_{m,n}\int_{f_{0}-B/2}^{f_{0}+B/2}[C_{mn}\left(f\right)E_{m,n}^{eign}\left(x,y,f\right)\\ e^{-\alpha_{mn}z}S\left(f\right)\delta \left(t-\frac{z}{v_{m}n}\left(f\right)\right)\cos\left(2\pi ft-\beta_{mn}z\right)]df\} \end{array}$$

where

(27) $$\delta \left(x\right)=\left\{\begin{array}{cc} 1, & ifx\geq 0\\ 0, & otherwise \end{array}\right.$$

### 4.5. Sub-Gallery Room-and-Pillar Model

The interior area of a mine is usually large and irregular, which causes reflection for the transmitted signals. The impact of reflected signals from the vertical sidewalls is nominal but the reflection due to the ceiling/floor is significant. A specific approach is adopted in this study to model this structure as a planar air waveguide and the same procedure is used to compile a multimode model as used in the mine gallery [30].

It is observed that the scenario of images and reflected rays are still considerable here, as only the *y*-coordinate is affected, and in this case, the incident angle is observed to be 0°. The reflection coefficient is $\left(1-\sqrt{k_{h}}\right)/\left(1+\sqrt{k_{h}}\right)$ for the distributed field of *X*-polarized and $\left(\sqrt{k_{h}}-1\right)/\left(\sqrt{k_{h}}+1\right)$ for the field of *Y*-polarized. If the transmitting node and reception node is placed at the height of $y_{0}$ and *y*, respectively, then the EM field at the reception node can be computed as

(28) $$E^{Rx}=E_{0}\sum_{q}\left[\frac{exp(-jky_{q}\left(y\right))}{y_{q}\left(y\right)}\right]{\left(\frac{1-\sqrt{k_{h}}}{1+\sqrt{k_{h}}}\right)}^{\left|q\right|}$$

where $y_{q}\left(y\right)$ denotes the distance between the reception node and $I_{q}$. The following expression can be used to calculate the value of $y_{q}\left(y\right)$

(29) $$y_{q}\left(y\right)=\left\{\begin{array}{cc} |2qb-y_{0}-y|, & \mathrm{if}\;q\;\mathrm{is}\;\mathrm{odd}\\ |2qb+y_{0}-y|, & \mathrm{if}\;q\;\mathrm{is}\;\mathrm{even} \end{array}\right.$$

Please note that the Eigen-function for *X*-polarized modes is obtained from [34]. The mode intensity $C_{n}$ is simplified from Equation (28) using the Poisson sum method and the expressions are given below

(30) $$E_{n}^{x}\left(y\right)=E_{0}.\cos\left[\left(\frac{n\pi }{2b}-j\frac{n\pi }{2b^{2}k}\frac{k_{h}}{\sqrt{k_{h}-1}}\right)y+\varphi_{y}\right]≅E_{0}\cos\frac{n\pi }{2b}y+\varphi_{y}$$

where $\varphi_{y}=\pi /2$ if *n* is even; $\varphi_{y}=0$ if *n* is odd.

(31) $$C_{n}\left(z\right)=\frac{E_{0}\pi }{bz\sqrt{1-{\left(\frac{n\pi }{2bk}\right)}^{2}}}\cos\left(\frac{n\pi }{2b}y_{0}+\varphi_{y}\right)$$

Now the field at any point can be calculated by computing the Eigen-function and intensity of each mode.

## 5. Results and Analysis

This section discusses the evaluation parameters that are considered to analyze the effectiveness of the suggested hybrid model and the influence of wireless link factors for an underground coal mine. Multiple evaluating parameters are analyzed and described briefly before presenting the simulated results. A detailed discussion is given for the graphical results of these parameters. Table 2 shows the values of system parameters used for the analysis.

### 5.1. Path Loss

The non-uniform structure, rough boundary surfaces and volatile environment of a mine intrudes the propagated signal and causes severe propagation loss. The reception power of the signal relies on the frequency and link distance covered by the propagated signal. Therefore, frequency and distance-dependent path loss is simulated. Mathematical expression to compute path loss and its analysis is discussed below. The path loss can be computed by taking the impulse response of a complex channel and the simplified expressions are given below [34]

(32) $$\begin{array}{c} PL{\left(z\right)}_{dB}=-10log_{10}{\left(\frac{1}{N}\sum_{j=1}^{N}\left|H\left(f,z\right)\right|\right)}^{2} \end{array}$$

(33) $$\begin{array}{c} P(f,z)=PL(f)PL(z) \end{array}$$

where PL(f) is frequency-based path loss and PL(z) represents the distance-dependent path-loss parameter and the generic function of integrated path loss is given in Equation (32). Equation (33) is extended for specific expressions of frequency-dependent and distance-dependent path loss, as [35]

(34) $$\begin{array}{c} PL\left(f\right)\propto ke^{\left(-\delta_{1}f\right)} \end{array}$$

(35) $$\begin{array}{c} PL_{dB}=PL_{dB}\left(d_{0}\right)+10nlog\left(\frac{z}{d_{0}}\right)+X\sigma \end{array}$$

where $PL_{dB}\left(d_{0}\right)$, *n*, and Xσ represents loss at reference distance, exponent for propagation loss, and fading parameter with standard deviation σ, respectively.

**Figure 5:**
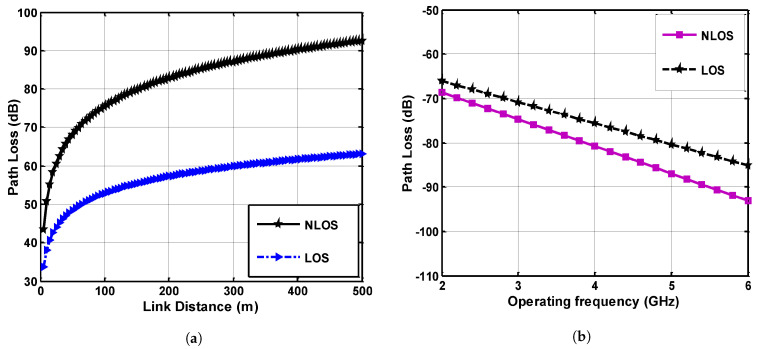
Path-loss analysis, (**a**) Distance-based path loss, (**b**) Frequency-based path loss.

Graphical results for path loss are given in Figure 5 which shows the relationship between the path loss and link distance for the LOS path and non-line-of-sight (NLOS) channel link. It can be observed from Equation (35) that the value of path-loss exponent *n* is slightly higher for the NLOS case where signal power is received through specular reflection which enhances the overall propagation loss. Figure 5a shows that the path-loss difference between LOS and NLOS link is comparatively low in the near region due to the low attenuating effect, but as the distance increases, the response of path loss increases exponentially. The path loss at all frequencies attenuates fast in the beginning because in the near region, higher modes with high attenuation rates exist, while in the far region lower modes dominate. Distance dependency in both the cases attenuates the signal, and this behavior clarifies the direct relationship of propagated signal performance with the link distance.

Neglecting the negative sign of frequency-dependent path loss in Figure 5b, the behavior of path loss is very clear regarding the frequency response of signals. The range of frequency is considered from 2 GHz to 6 GHz, but the optimal use of frequency for this slot is 2.4 GHz and 5 GHz, hence path-loss response at these two particular frequencies is investigated by classifying LOS links for different segments of the mine. It can be seen that at the initial values of slot 2.4 GHz, the obtained path loss is low as compared to the higher frequency of this band. The comparative difference between both links is low at 2.4 GHz, but as the operating frequency is increased to 5 GHz, the path-loss difference is highly increased.

### 5.2. Received Power

Signal power plays an important role in signal propagation and medium characteristics in which the signal is traveling between transceiver nodes. The critical characteristics of mines induce a negative impact on signal transmission resulting in low power reception. Evaluating signal power at the reception point is a basic approach to estimate the condition of data extraction from the received signal. The multimode model is implemented in this study to derive Equation (23) for received power in the particular room-and-pillar environment of the mine. The comparative analysis of the transmitted signal at 2.4 GHz and 5 GHz frequency is performed for the link distance of 500 m. Moreover, the received power response is examined by classifying LOS zones.

**Figure 6:**
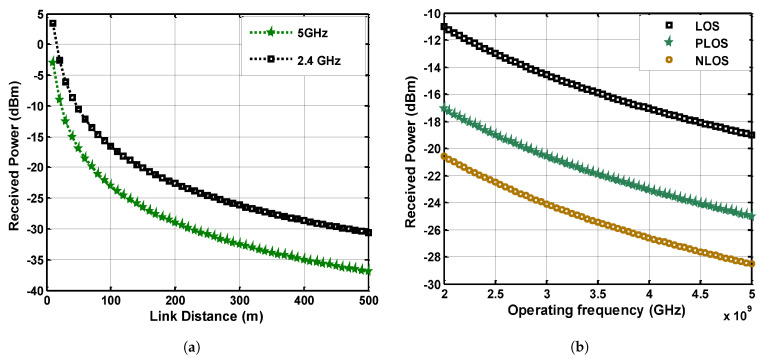
(**a**) Received power comparison at 2.4 GHz and 5 GHz, (**b**) Received power analysis at 2 GHz–5 GHz band for LOS, NLOS and PLOS links.

Figure 6a shows the comparative analysis of received power between 2.4 GHz and 5 GHz. The received power is calculated at multiple frequencies and particularly the response is much better at 2.4 GHz as compared to 5 GHz. It depicts that the attenuating factor has a mild effect at lower frequencies of the UHF band in mines, which leads to better received power at the reception point. It can be seen that the power of the signal is declining exponentially at the beginning, but the received power response becomes linear with the rise of link distance. The major effect of attenuation on the propagated signal can be mitigated to some extent by the hike of transmission power.

Similarly, signal propagation analysis is taken in a mine’s sub-gallery where the LOS path is broken and classified into LOS, NLOS, and partial line-of-sight (PLOS) segments. The received signal power is calculated for given LOS categories and the results are given in Figure 6. It can be observed from Figure 6b that there is a clear difference in the signal-received power. The received power for LOS is much higher than NLOS and PLOS links, and the response of the analysis concerning frequencies comparison is nearly constant throughout the link distance. The difference in signal power of PLOS and NLOS is not considerably high but for a clear LOS link, the performance of communication is much better.

**Figure 7:**
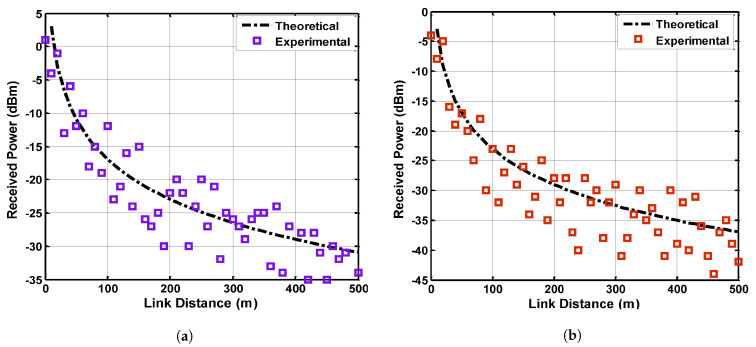
Received power comparison between theoretical and experimental results (**a**) 2.4 GHz, (**b**) 5 GHz.

Performance analysis of received power is carried out using the experimental data to validate the theoretical received power, as shown in Figure 7. Long-range measurement is required to examine the characteristic behavior of EM-propagated signals in the environment of an underground mine. The experiments are taken at 2.4 GHz and 5 GHz frequency band in the mine gallery (coal mine, Xian, China) which has 600 m length, 5 m width and 4 m height. The mine contains mining equipment and infrastructure. The transmitters are mounted at multiple locations such as ceilings, walls, and in the center of the gallery at a height of 3 m, 2.5 m, and 2 m, respectively. The receivers are oriented at the same height, and both transmitters and receivers are equipped with Omni-directional antennas (EM 6116). The antennas are vertically polarized and support the frequency band of 2 GHz to 5 GHz. The gain of the antennas and the permittivity values of the environmental material are described in Table 2.

The spatial variations of the channel are determined by moving the transmitting antenna on a rectangular grid of 7 × 5 by considering a static channel environment and fixed receiver. The grid spacing is considered to be 7 cm for the operating frequency of 2.5 GHz and 5 GHz to obtain the independent samples. The effect of the mobile receiver is captured as well, between link distances of 10 to 500 m for fixed transmitters. The transfer function of the channel and received signal power is measured using a Vector Network Analyzer (VNA HP8753ES) and Matlab, as shown in Figure 4a. Each transfer function is obtained for the frequency bands of 2.4 GHz to 5 GHz by taking 1601 frequency gap points with the spectral resolution of 1.87 MHz. The measurements taken in frequency response are easier for calibration under the conditions of a static environment. The low-loss coaxial cables are used with the transmitting and receiving antenna. The calibration for the attenuation of connectors and loss due to coaxial cable is considered while computing the received signal power.

The transmitting antennas are located at positions (2, 3, 3), (0.5, 2.5, 5) and (2.5, 2, 7) and the receiving antennas are placed at (2, 3, 10), (0.5, 2.5, 50), and (2.5, 2, 100). The measurements are carried out for the range of link distance from 10 to 500 m for LOS and NLOS cases by splitting the area of the gallery due to the non-uniform structure of the mine. A set of three measurements are taken for each link distance by varying the transmitter positions, and after taking these values, the receiving antenna is switched to another location for the consideration of the next three values. The antenna polarizations at corresponding locations are taken carefully against the propagation loss and the link distance is varied up to 500 m for the collection of the measured data. The channel of the propagated signals is considered stationary during the measurement, and the received signal power is collected from multiple locations including the propagation loss and SNR. Figure 7 shows the comparison analysis of theoretical and experimental received power at 2.4 GHz and 5 GHz. It can be seen that the behavior of the theoretical received power is nearly accurate even for the different operating frequencies. A minor deviation of approximately 12 to 16% can be noticed which validates the predicted received power values by the suggested approach.

### 5.3. Root Mean Square Delay Spread

The time delay between multiple reflected waves is another important parameter to consider while characterizing the multipath channel in underground mines. It is used to determine the frequency-based degradation in the erratic environment of mines, where the signal is facing a lot of deteriorating factors to disperse it into multiple paths. It restricts the data rate of the wireless link during the transmission of signals in mines. It is computed from the PDP expression derived in Equation (26) and the generic model is expressed in Equations (36)–(38) [29,36]:

(36) $$\begin{array}{c} \tau_{rms}=\sqrt{\bar{\tau^{2}}-{\bar{\tau }}^{2}} \end{array}$$

(37) $$\begin{array}{c} \bar{\tau }=\frac{\sum_{k}a_{k}^{2}\tau_{k}}{\sum_{k}a_{k}^{2}}=\frac{\sum_{k}p\left(\tau_{k}\right)\tau_{k}}{\sum_{k}p\left(\tau_{k}\right)} \end{array}$$

(38) $$\begin{array}{c} {\bar{\tau }}^{2}=\frac{\sum_{k}a_{k}^{2}{\tau_{k}}^{2}}{\sum_{k}a_{k}^{2}}=\frac{\sum_{k}p\left(\tau_{k}\right){\tau_{k}}^{2}}{\sum_{k}p\left(\tau_{k}\right)} \end{array}$$

where $a_{k}$ is the gain coefficient, $p(\tau_{k})$ is the PDP of the $k^{th}$ multipath component and $T_{k}$ is the delay coefficient.

**Figure 8:**
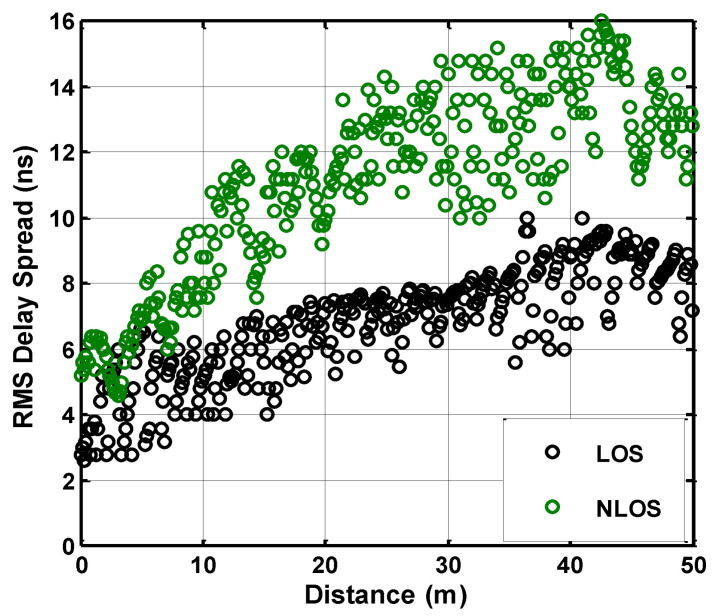
Root mean square delay spread response.

The RMS value is computed for mine sub-gallery where the link is classified into LOS and NLOS cases. The RMS delay spread is computed at multiple locations in the sub-gallery using the threshold level 10 dB. Figure 8 shows the graphical result and it can be observed that the value of RMS delay spread rises to a certain limit in the case of distant reception nodes and then it gradually starts decreasing. The maximum-to-minimum variation captured in RMS delay spread is 2.40 ns to 10 ns for the LOS link and 4.20 ns to 15.95 ns for the NLOS, respectively. The values of RMS delay spread are found to be higher for occupied mine galleries due to the existence of mining equipment and pillar structure. It indicates that the signal propagation over the NLOS link must face more spatial variation than the LOS link due to the presence of more scattering objects. The propagated waves take an elongated path due to the severe number of reflections caused by the mine environment, which will further enhance the delay at reception. The irregularity in the simulated graph depicts the unevenness of mine walls, which generates non-uniform reflected propagation.

### 5.4. Refraction Loss

The internal shape of underground mines and dimensions are rough enough to disrupt the originality of signal and the dielectric medium of mines badly affects the characteristics of signal transmission. Non-uniformity in the medium of mines and refraction by the floor and walls are regarded as refraction loss. Due to the penetration of transmitted signals through any medium, a portion of the signal may be absorbed, resulting in severe refraction loss.

Refraction loss is calculated by considering both horizontal and vertical medium characteristics, depending on the width and height of a mine. Only the dominant polarization is considered. The defined expression of refraction loss is given in Equations (39) and (40) [7,37]:

(39) $$\begin{array}{c} L_{H}=4.343\lambda^{2}\left(\frac{\varepsilon_{v}}{w^{3}\sqrt{\left(\epsilon_{v}-1\right)}}+\frac{1}{h^{3}\sqrt{\left(\varepsilon_{h}-1\right)}}\right)z \end{array}$$

(40) $$\begin{array}{c} L_{v}=4.343\lambda^{2}\left(\frac{1}{w^{3}\sqrt{\left(\varepsilon_{v}-1\right)}}+\frac{\varepsilon_{h}}{h^{3}\sqrt{\left(\varepsilon_{h}-1\right)}}\right)z \end{array}$$

where λ, *w*, *h*, *z*, $\varepsilon_{v}$, and $\varepsilon_{h}$ represents the frequency, width of mine, height, distance, the permittivity of sidewalls, and top ceiling, respectively.

**Figure 9:**
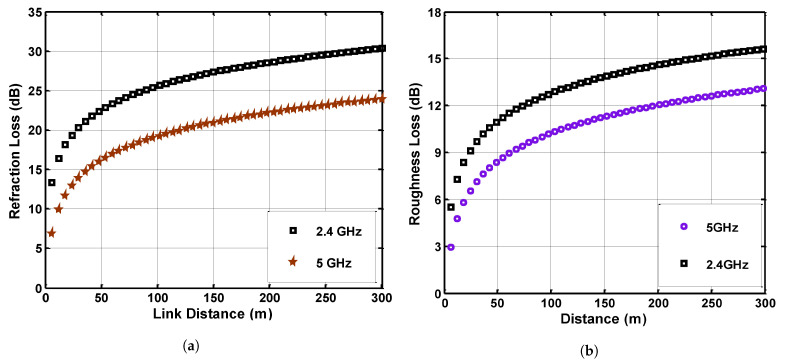
(**a**) Refraction loss at operating frequency of 2.4 GHz and 5 GHz, (**b**) Roughness loss at 2.4 GHz and 5 GHz.

The refraction loss is computed against horizontal and vertical polarization. The resultant sum is displayed in Figure 9 at the operating frequencies of 2.4 GHz and 5 GHz. Figure 9a shows the refraction loss for various link distances using different operating frequencies. There is an exponential rise in the refraction loss as the link distance increases due to the interior surface and room-pillar structure of mines. A higher loss is observed at a higher link range due to a directly proportional relationship with link distance. The relative permittivity of the medium has a minor effect on refraction, but the width and height of mine galleries is an important parameter to consider when measuring refraction loss. If the width and height of the gallery are low, there is a high probability of refraction. The operating frequency signal of 5 GHz is refracting less in the mine environment than that of 2.4 GHz.

### 5.5. Roughness Loss

The irregular variation on the surface of mines causes roughness, and such an uneven surface has a great influence on the propagated electromagnetic signal. The roughness of side walls causes signal scattering, which results in a deterioration in link quality. The expression of roughness loss for the mine gallery is given as [7,37]:

(41) $$L_{R}=4.343\pi^{2}r^{2}\lambda \left(\frac{1}{2w^{4}}+\frac{1}{2h^{4}}\right)z$$

where *r* is the Gaussian distributed root mean square roughness parameter. Figure 9b shows the roughness loss analysis under the operating frequency of 2.4 GHz and 5 GHz. The result clearly shows that roughness loss has a direct relationship with the link distance, and the response of roughness loss increases with the increase in link distance. Furthermore, it can be observed that the 5 GHz frequency is experiencing less roughness loss as compared to 2.4 GHz.

### 5.6. Bit Error Rate and Signal-to-Noise Ratio

A network link can be examined by evaluating its bit error rate (BER) and signal-to-noise ratio (SNR). For a simple Gaussian noise channel model, a propagated signal attains the noise from the medium through which signal is penetrating and, at the receiver end, the ratio of the original to altered bits is the key indicator of the quality of communication. The SNR for the wireless link in the underground mine is computed as the function of distance, and the expression is given in Equation (42) [38]

(42) $$SNR\left(z\right)=P_{T}+G_{T}+G_{R}-{\bar{PL}}_{dB}-NP$$

where ${\bar{PL}}_{dB}$ is the distance-dependent propagation loss and NP is the noise. After computing the SNR for the mine’s sub-gallery, the mine LOS segments are evaluated by calculating the BER. A data rate of 4800 bps is used to compute the BER by employing Binary Phase-Shift Keying (BPSK) modulation. Please note that the parameters in Equation (42) are different for each LOS classified link, including path loss and fading. The mathematical equation to calculate BER from SNR using BPSK is stated below in Equation (43)

(43) $$BER_{BPSK}=Q\left(\sqrt{2\frac{E_{b}}{N_{0}}}\right)\;;\;Q\left(z\right)=\frac{1}{2\pi }\int_{x}^{\infty }e^{-\frac{y^{2}}{2}}dy$$

**Figure 10:**
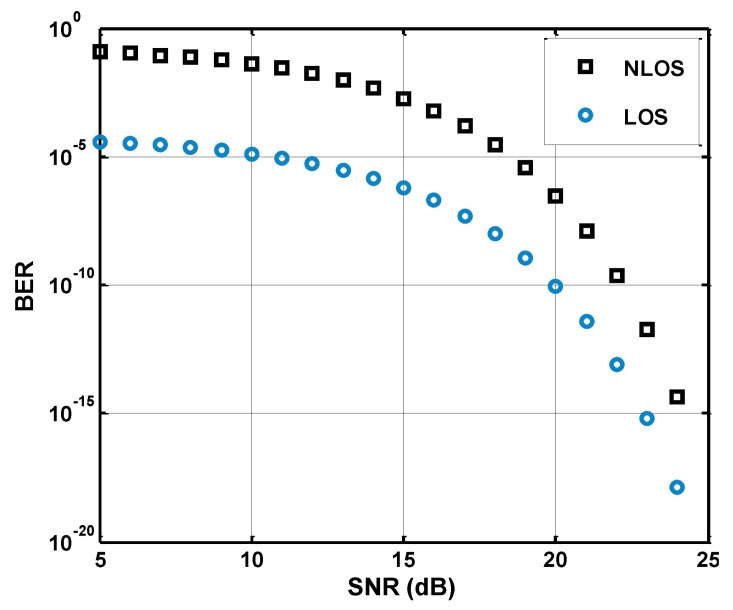
Signal-to-noise ratio and bit error rate analysis for LOS, and NLOS links.

Figure 10 shows the comparative analysis in terms of LOS segments. The SNR is computed using Equation (42), and it can be seen that a distance-dependent path-loss factor exists in SNR expression. The path loss is computed distinctly for each LOS link including fading. Therefore, it is clear that high link distance gives low SNR value, and for low SNR, the BER performance will be obtained with a high error value. It can also be observed that the NLOS link is running with the highest BER, whereas the LOS link is facing a low probability of error and the BER of PLOS is expected to be in the middle of both the links.

### 5.7. Shadow Fading and Signal Attenuation

It can be observed that in the case of a mine sub-gallery, the typical pillars of irregular dimension and the random distribution of pillars is a source of reflection and diffraction for propagated signals. Each mode experiences independent and distributed shadow fading, as a single mode comprises a bunch of rays with an identical angle. Therefore, the net field at any location can be obtained by adding up the field of all modes [29], as given below in Equation (44)

(44) $$E_{rx}\left(y,z\right)=E_{0}\sum_{n}C_{n}\left(z\right)E_{n}^{x}\left(y\right)e^{(-\alpha_{n}+j\beta_{n})z}X_{n}$$

where $X_{n}$ is an identically distributed and independent lognormal random variable; the approximate equations for $\alpha_{n}$ and $\beta_{n}$ are given as

(45) $$\alpha_{n}=\frac{1}{b}{\left(\frac{n\pi }{2bk}\right)}^{2}Re\frac{1}{\sqrt{\bar{k_{h}}-1}}\;;\;\beta_{n}=\sqrt{k^{2}-{\left(\frac{n\pi }{2b}\right)}^{2}}$$

By compiling distance-dependent path loss from Equation (35) and the shadow-fading parameter from Equation (44), a relationship can be analyzed between shadow fading and path loss.

**Figure 11:**
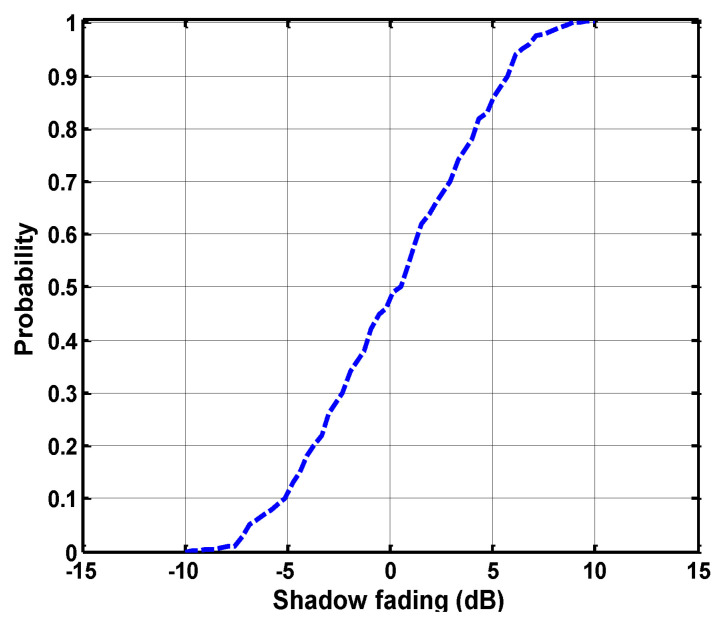
Cumulative distribution function of shadow fading.

The shadow-fading parameters vary in different zones of the mine, and this variation shows a lognormal distribution. The cumulative distribution function (CDF) is drawn, and the probability of its deviation is shown in Figure 11. It can be observed that obstructions in the internal congested mine galleries and rough non-uniform structure creates a heavy signal-deteriorating effect for propagation. The attenuation coefficients are measured by considering the attenuation of each mode of every field, as elaborated in Equations (7) and (45).

## 6. Conclusions

This study approaches the problem of efficient channel modeling for the adverse and erratic environment of underground coal mines. It reveals that different areas of an underground mine need to be characterized separately for electromagnetic signals. It is very important to efficiently model the entire environment of a mine for the development of a stable wireless network. The areas of a mine are classified into main gallery and sub-galleries, and a hybrid model scheme is used for channel modeling of complex mines. The EM wave propagation characteristics are defined by the multimode model, and field intensities are estimated with the aid of the GO model. The received power and various evaluating parameters are computed to examine the effectiveness of the suggested model. The complex segments and branches of the mine are classified by defining the line-of-sight, non-line-of-sight, and partial-line-of-sight zones for managing the optimized signal strength in every zone of an underground mine. The environment of the mine rapidly changes due to mining activities, and the materials that occupied the mine are a big source of deterioration for wireless communication. A detailed evaluation of these degrading elements is conducted in this research, which elaborates that the irregular structure of a mine, the roughness of its boundary walls, the dielectric medium of a mine, and equipment used for mining applications cause major disruption for signal propagation and network decay. Operating frequency is the most important parameter in a wireless network and, after a detailed examination of 2 GHz to 5 GHz band frequencies in different zones of the underground mine, it can be concluded that a lower-frequency signal in the UHF band attains low path loss, but captures high refraction loss and roughness loss. The overall performance of the 2.4 GHz frequency is much better than the 5 GHz frequency in the harsh environment of underground coal mines.

## Figures and Tables

**Table 1:** Summary of advantages and disadvantages of existing communication channel models for underground mines.

**Models**	**Advantages**	**Disadvantages**
Single mode	Simple computation	Invalid for high frequencies
	Considers Physical factors	Only path-loss evaluation
	Frequently used in theoretical models	Invalid for near zone
GO model	Simplicity	Less accurate on low frequencies
	Considers tunnel branches	Complex computation on high link distance
	Considers physical factors	
Multimode	Valid for multiple frequencies	Complex
	Valid for near & far zone	Incapable for wall roughness
	Valid for RMS delay	Invalid for mine branches
Full-wave	High accuracy	Complex
	Capable for wall roughness and branches	FDTD computational extension

**Table 2:** Particular and associate values used for simulations.

Parameter	Symbol	Values
Transmitted Power	$P_{T}$	13.97, 16.98, 18.75 dBm
Transmitter Gain	$G_{T}$	2 dB
Receiver Gain	$G_{R}$	2 dB
Operating frequency	*f*	2.4 GHz–5 GHz
Link Distance	*z*	0–500 m
Width of Mine gallery	*w*	5 m
Height of Mine gallery	*h*	4 m
Attenuation Coefficient	$\alpha_{n}$	10–20 dB/m
Vertical walls permittivity	$\varepsilon_{v}$	5 $\varepsilon_{0}$
Horizontal permittivity	$\varepsilon_{h}$	4 $\varepsilon_{0}$
Wavelength	λ	0.1249, 0.0599 m

## Data Availability

Not applicable.
